# Welcome to your *Journal of Clinical and Translational Science*

**DOI:** 10.1017/cts.2017.3

**Published:** 2017-04-12

**Authors:** Arthur M. Feldman

**Affiliations:** Department of Medicine, Lewis Katz School of Medicine at Temple University, Philadelphia, PA 19140, USA

Over the past 2 years, numerous members of the Association of Clinical and Translational Science (ACTS) have worked assiduously along with members of the editorial team at Cambridge University Press to launch the *Journal of Clinical and Translational Science* (JCTS). This new journal is not a recapitulation of our previous journal, Clinical and Translational Science, but rather a new journal with new goals and a new mission. Some of the differences can be seen in the advertising that you should shortly see in your in-box and some are less apparent. Therefore, let me elaborate. First and foremost, JCTS belongs to the ACTS. It will have the editorial freedom afforded all academic journals; however, the Association, through its Publication Committee, will oversee the activities of the journal; the ACTS will share in any positive margins accrued by the journal once the costs are met, and ACTS members and K scholars will be able to take advantage of special programs. Importantly, the structure of the Editorial Board and the diverse interests of its members point to a primary goal for the journal: to publish outstanding research in all areas of translational science—T1 to T4.

The goal of becoming a top-flight journal in the many aspects of translational research is audacious; however, we believe that the goal is achievable. First, there are important areas of translational research that do not have natural editorial homes: for example, health disparities research, community engagement research, and population health research. Second, some journals have become narrowly focused and/or Editorial Boards have become protective of areas of research that now occupy members of the translational science community—education of the translational science workforce being one example. Third, some journals are afraid to take on the tough issues that face the translational sciences. Finally, the enormous volume of manuscripts submitted from around the world to US journals has far outpaced the number of manuscripts that the existing high-quality journals in aggregate can accommodate. Therefore, many excellent papers are being left without a home. However, in order to rise to the top, we have to provide services that are both competitive and innovative. Therefore, we have taken the following steps:

1. We will accept cascaded peer reviews for papers rejected by other journals. Authors must provide a point-by-point rebuttal to the reviewers’ criticisms and/or have, where appropriate, performed an additional experiment or experiments to address the reviewer’s comments. An editorial-level review may be performed; however, JTCS reserves the right to send the paper for additional review.
2. When a K Scholar is the first author of a manuscript, the K Scholar will have the opportunity to speak with the Editor or with the Deputy Editor who handled the paper after receiving the reviewer’s comments and the Editor’s decision, so that they can fully understand exactly what needs to be done to make the paper acceptable for JCTS.
3. Whenever possible, we will structure responses to the authors that specifically dictate those changes or new experiments that are required to make the manuscript acceptable. This will be an attempt to eliminate the sometimes interminable back and forth between authors and reviewers.
4. We have expanded and reorganized the board with Deputy Editors and Senior Editors for: (1) Basic Translational Research, (2) Clinical Research, (3) Education, (4) Population Health, Community Engagement, Health Disparities & Health Policy, and Translational Research Design and Analysis. We believe that this outstanding group of translational scientists will provide fair and helpful reviews in their areas of expertise and help attract excellent papers. As we move forward, we will certainly need to expand our Senior Editor group and form an Editorial Board.
5. With the help of Cambridge University Press, we hope to obtain PubMed indexing at the earliest possible time; however, in the interim, we will ensure that all papers that report work that was supported by National Institutes of Health grants will be submitted to PubMed Central and indexed in PubMed in a timely manner.
6. All manuscripts will be published online in a timely manner and will appear with their final citation details.
7. Finally, we intend to distribute the Table of Contents for each issue as widely as possible as well as taking advantage of social media to help publicize the findings of significant papers to the global scientific community.

Most importantly, however, JCTS is now YOUR journal. We have provided opportunities for members of the translational science community to provide opinions through letters, brief reports, and review articles. By publishing in JCTS, you will actually be able to support the Association and all of its activities that benefit both investigators and trainees. Moreover, as the journal grows, we hope that many of you will agree to add your names to the Editorial Board and to the group of Senior Editors. This is an exciting time for JCTS and for the ACTS, and I am honored to serve as Editor. I look forward to working with all of you as we bring the vision for JCTS to a reality.

